# Relationships between Rainy Days, Mean Daily Intensity, and Seasonal Rainfall over the Koyna Catchment during 1961–2005

**DOI:** 10.1100/2012/894313

**Published:** 2012-05-02

**Authors:** S. Nandargi, S. S. Mulye

**Affiliations:** Climatology and Hydrometeorology Division, Indian Institute of Tropical Meteorology, Dr. Homi Bhabha Road, Pashan, Pune 411 008, India

## Abstract

There are limitations in using monthly rainfall totals in studies of rainfall climatology as well as in hydrological and agricultural investigations. Variations in rainfall may be considered to result from frequency changes in the daily rainfall of the respective regime. In the present study, daily rainfall data of the stations inside the Koyna catchment has been analysed for the period of 1961–2005 to understand the relationship between the rain and rainy days, mean daily intensity (MDI) and seasonal rainfall over the catchment on monthly as well as seasonal scale. Considering the topographical location of the catchment, analysis of seasonal rainfall data of 8 stations suggests that a linear relationship fits better than the logarithmic relationship in the case of seasonal rainfall versus mean daily intensity. So far as seasonal rainfall versus number of rainy days is considered, the logarithmic relationship is found to be better.

## 1. Introduction

The amount of rainfall received over an area or a basin is an important factor in assessing the amount of water available to meet the various demands of agriculture, industry, irrigation, hydroelectric power generation, and other human activities. The distribution of rainfall in time and space, therefore, is an important factor in determining the economical status of a region, or a state or a nation. Much of the information about the rainfall climatology of any region or a basin is mostly based on monthly, seasonal, and annual rainfall data that are derived from daily rainfall recorded at individual stations. Two relatively simple parameters which assist in providing a better picture of rainfall conditions than does the monthly total alone are number of rain days and mean daily rainfall intensity. They give some indication of frequency of occurrence and a crude measure of intensity of rain; both of these characteristics have agricultural and hydrological significance.

 A number of studies on these two elements have been carried out for various parts of the world, including their relationships with the seasonal totals. Olascoaga [1] made a rainfall—rain day study of 10 regions in Argentina utilizing daily rainfall data for 5 years. He found that a single normalized rainfall curve gave a satisfactory representation of the rainfall distribution of all the rainfall regions. Similar conclusions were made by Rai Sircar [2] from a study of the south-west monsoon rainfall of groups of stations around Delhi, Calcutta, Bangalore, and Tiruchirapalli. A summary of these and other studies of a similar nature has been given by Riehl [3, 4] in his well-known books.

 The other studies include analyses by Jackson [5] for Tanzania and Harrison [6] for South Africa. These studies examine the nature of the relationships and the deviations from the relationships for individual stations and regions. Jackson [7, 8] suggested that rainfall studies of a wide range of shorter period are extremely important. By analyzing the data for a range of tropical rainfall stations, he also showed that a better relationship exists between monthly rainfall and number of rain days than between monthly rainfall and mean daily rainfall intensity.

 Ananthakrishnan and Soman [9] made a detailed study of the normalized rainfall curve (NRC) by utilizing the daily, monthly, seasonal, and annual rainfall data of 15 Indian stations covering wide variety of rainfall regions for the 80-year period from 1901 to 1980. They showed that the daily rainfall distribution can be delineated by a normalized rainfall curve (NRC). However, there is no universal normalized curve which can represent all rainfall regimes as suggested in some of the earlier studies.

 From the foregoing studies, it is understood that the variations in the frequency of, or rainfall amounts on the heavier rainfall days may, therefore, be expected to exert a major control on rainfall totals. The rain days study can provide the information regarding frequency and intensity of rain events during different weather conditions. For example, drought season may be marked by both fewer rain days and less rain per day as compared to periods of normal and above-normal rainfall. Therefore, statistical features of the daily rainfall distribution at different stations over an extended area are interesting and important aspects in rainfall climatology and hydropower generation. In view of this, an attempt has been made to study the intensity, frequency, and duration of rainfall with reference to number of rain and rainy days for the period of 1961 to 2005 over the Koyna catchment up to Koyna dam site (*hereafter referred to as Koyna catchment*) in Satara district which is one of the well-known hydropower generating centre in the peninsular India.

The Koyna catchment in India is always in discussion of geoscientists as the area comes under the high seismic activity. After an earthquake of magnitude 7 in 1967, detailed geological, tectonic, and seismic investigations of this river basin have been carried out by several workers. However, it is also interesting and essential to study the rainfall characteristics of the Koyna catchment from hydro-geological point of view and to understand the impact of climate change after the construction of the dam in 1963 with special reference to rainfall intensity, rainy days, and so forth.

## 2. Study Area

**Figure 1:**
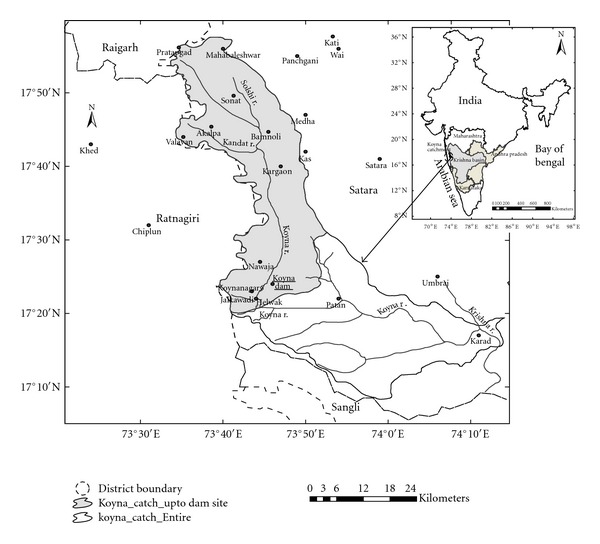
Illustrated map of the Koyna catchment in the Peninsular India.

The Koyna catchment is located in the Western Ghats of Maharastra state in Satara district approximately between the latitudes 17°20′N and 17°55′N and longitudes 73°35′E and 73°55′E (see Figure 1). The catchment is parallel to the Western Ghats for about 70 km in length with about 12 to 13 km in width. Spurs of the Western Ghats stretch into the catchment from the west playing important role in the distribution of the rainfall.

The Koyna River rises in Mahabaleshwar (viz. Malcompeth Plateau) on the Western Ghats and is a major tributary of the Krishna River in western Maharashtra. The river is just about 100 m in width and is slow flowing. Unlike most of the other rivers in Maharashtra which flow East-West direction, the Koyna river flows in North-South direction for about 65 kms, turns sharply near Helwak, and joins the Krishna River near Karad, which is one of the three largest rivers in southern peninsular India (see Figure 1). The River is famous for the Koyna Dam which is the largest hydroelectric project in Maharashtra. The reservoir known as “Shivaji Sagar Lake” is a huge lake of 50 km in length. The dam is situated in Koyna Nagar in the Western Ghats and is built in 1963.

An attempt has been made in the present study to understand the rainfall characteristics over the Koyna catchment up to Koyna dam site, since the construction of dam in 1963, especially from climate change point of view, with the following objects:

- to study heavy rainfall distribution over the Koyna catchment and its variation in different decades 1961 to 2005,
- identify the days of heavy rains,
- relative importance of rain day frequency and intensity changes on monthly and seasonal scale,
- the upper limit of rain intensity (mm/day) contributing 50% of the seasonal rainfall,
- estimation of rain intensity (mm/day) at 99% and 99.9% of the cumulated number of rain days,
- the difference between the number of “rain-days” and “rainy days” expressed as a percentage of the number of rain-days,
- to study relationship between seasonal rainfall, rainy days and mean daily intensity (MDI).

## 3. Data Used

In the Koyna catchment (up to dam site) (see Figure 1) there are 8 stations, namely, Pratapgad, Mahabaleshwar, Valvan, Bamnoli, Kargaon, Sonat, Navaja, and Koynanagar (Koyna) whose daily rainfall data for the monsoon season (June to October) for the period from 1961 to 2005 have been considered in the present study as most of the rainfall is received during this season. Of these stations, Mahabaleshwar rain gauge station is maintained by India Meteorological Department (IMD) while other 7 stations are maintained by the Koyna dam maintenance authority. Mahabaleshwar is one of the oldest rain gauge stations in the Maharashtra state, and the data for the same is available since 1829. It may also be mentioned that all the rain gauge stations except Mahabaleshwar and Koynanagar were discontinued from 1973 and restarted since 1979. Hence, the daily rainfall data for 5 stations were not available for the period 1973 to 1978. Besides, Kargaon is a newly introduced station and started functioning from 1993 only.

## 4. Methodology

 A technique of analyzing the daily rainfall data that provides much insight into the nature of the rainfall distribution is the investigation of the association between the cumulated percentage rain amount and the cumulated percentage number of rain days (rain frequency) after arranging the rainfall data series in ascending or descending order of rain amount. In the present study, the term “rain day” is used to denote a day on which a station has recorded 0.1 mm or more rainfall. The term “rain event” is used to denote the occurrence of rain amount 0.1 mm in a specified interval of time and “rain intensity” to indicate the 24-hr rainfall amount (see Table 3).

 The daily rainfall data series of the eight stations located inside the catchment were assembled and analysed for (a) each of the monsoon month, (b) monsoon season as a whole comprising 6 rainfall data series for each of the 8 stations and a total of 48 rainfall series. The characteristics of the distribution of daily rainfall have been studied by examining the association between the cumulated percentage rain amount (*x*) and the cumulated percentage number of rain days (*y*) after arranging the rainfall series for a given period of season in an ascending sequence of daily rainfall. Here, the values of *x* and *y* range from 0 to 100 per cent in ascending order. The association between *x* and *y* is shown by a curve which is referred to as the normalized rainfall curve (NRC). The NRCs for all the stations can be represented by the analytical equation

(1) $$\begin{array}{c} x=y\exp\left[-b{\left(100-y\right)}^{c}\right], \end{array}$$

where

(2) $$\begin{array}{c} x_{k}=100\overset{k}{\underset{i=1}{\sum }}\frac{r_{i}}{R},\;k=1,2,\ldots N,\;y_{k}=100\left(\frac{k}{N}\right) \end{array},$$

  *b* and *c* are empirical constants that depend on the CV of the rainfall series.

Here, *R* = ∑_*i*=1_^*N*^ *r*_*i*_ = total  rain  amount and *r* = *R*/*N* = average  rainfall  per  rain  day.

 As *k* takes values from 0 to *N*, *x*_*k*_ and *y*_*k*_ take values from 0 to 100. Graphical representation of the corresponding sets of values (*x*, *y*) gives the normalized rainfall curve (NRC). The rain intensity (i.e., rain amount per day) corresponding to any point on the NRC is inversely proportional to the slope of the tangent to the curve at that point. The point on the NRC where the slope of the tangent is 45° corresponds to the mean daily rain amount *r* = *R*/*N*, where *R* is the cumulated total rain amount and *N* is the cumulated total number of rain days. Unlike the original equation for NRC, (1) can account for the high-intensity rainfall at the upper extremity of the NRC in accordance with observation.

Since the catchment is located in the hilly terrain, receiving heavy to very heavy rainfall throughout the monsoon season, an attempt has also been made to study the relationship between rain day and a rainy day. According to India Meteorological Department (IMD), a rainy day has been defined as a day with rainfall of 2.5 mm or more rainfall. IMD further defines that rainfall for a station is called heavy if it is greater than 650 mm and very heavy if it is greater than 1300 mm. Following these criteria, average numbers of rain days have been calculated for each of the monsoon month 1961 to 2005 period for the stations in the Koyna catchment.  Table 5 gives the average number of rain days for the stations in the Koyna catchment.

## 5. Characteristics of Seasonal Rainfall over the Koyna Catchment

**Figure 2:**
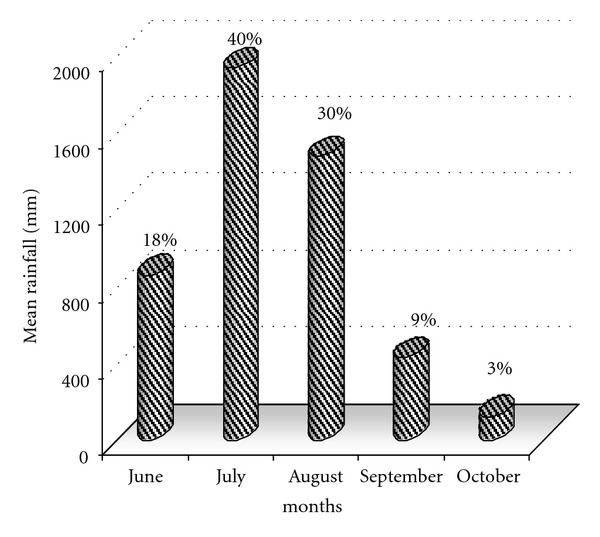
Monthly average rainfall (mm) over the Koyna catchment.

 On the basis of available daily rainfall data of all the eight stations for the period of 1961 to 2005, it is seen that the mean seasonal rainfall over the entire catchment is of the order of about 4824 mm, declining from 1940 mm in July to 120 mm in October. July and August are the two major contributing months to the annual rainfall (see Figure 2).

**Figure 3:**
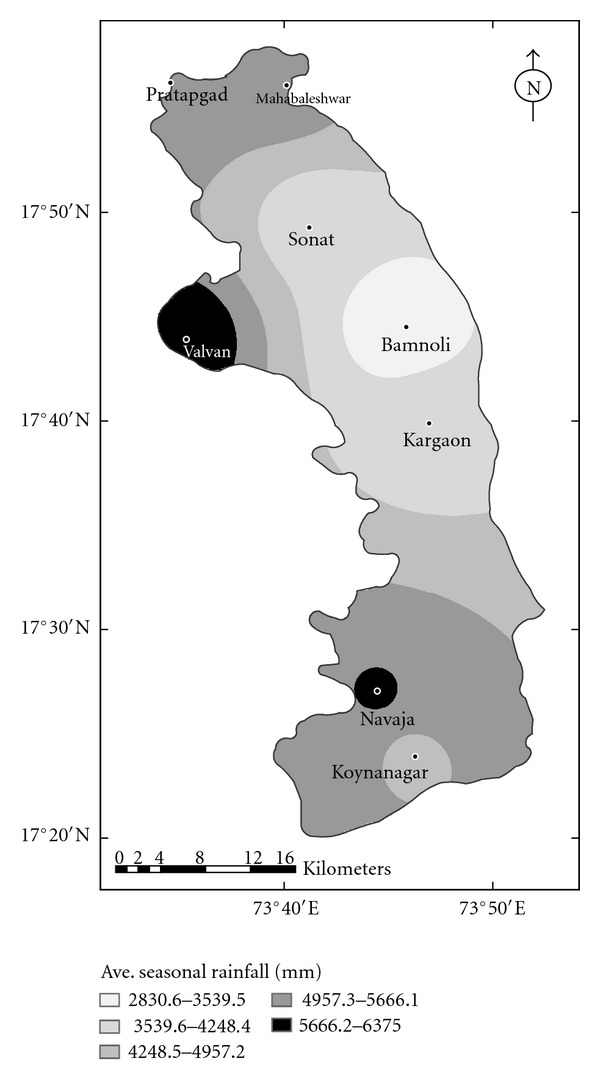
Spatial distribution of average seasonal rainfall (mm) over the Koyna catchment.

Nearly 70% of the seasonal rainfall occurred in these two months.  Figure 3 shows the spatial distribution of average seasonal rainfall over the Koyna catchment. It is seen that the heaviest seasonal rainfall occurs over the western parts of the catchment, and the lowest rainfall occurs over the eastern parts of the catchment. The mean annual rainfall of the catchment is highly correlated (+0.97) with the Mahabaleshwar annual rainfall [10], and the linear regression equation connecting the two is

(3) $$\begin{array}{cc} & \text{Mean}\;\text{annual}\;\text{rainfall}\;\text{of}\;\text{the}\;\text{Koyna}\;\text{catchment}\\ & =0.7981\left(\text{Annual}\;\text{rainfall}\;\text{of}\;\text{the}\;\text{Mahabaleshwar}\right)-17.445. \end{array}$$

Considering the Mahabaleshwar's mean annual rainfall (50 year normal) of 6226.3 mm and using (3), the mean annual rainfall of the Koyna catchment is estimated to be of the order of 4951.77 mm. About 95% of the annual rainfall occurred from June to September months and 97% during June to October months. It is essential to mention here that the monthly average rainfall for the Mahabaleshwar station when compared for the two periods, namely, 1901–1950 and 1951–2005 showed that there is a decrease in the monthly average rainfall during 1951–2005.

**Figure 4:**
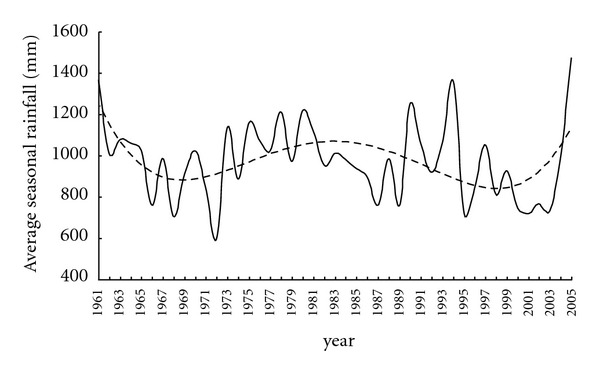
Yearly variation of average seasonal rainfall over the Koyna catchment.

**Figure 5:**
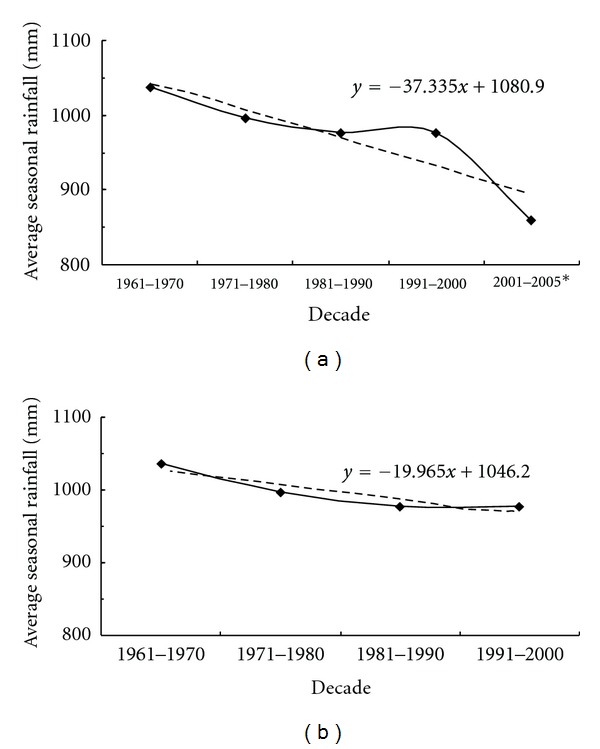
Decadal variation of average seasonal rainfall over the Koyna catchment.

 The average seasonal rainfall for each year shows that the rainfall was reduced during the decades of 1961 to 1970 and 1991 to 2000 (see Figure 4). It is also seen that rainfall activity has increased during the recent period of 2003 to 2005. However, the decadal variation of average seasonal rainfall shows a decreasing tendency (see Figure 5(a)) when the entire data period from 1961 to 2005 has been considered. If the decadal variation is seen only for the complete decades, namely, 1961–1970, 1971–1980, 1981–1990, and 1991–2000 (since, *2001–2005 is not a complete decade), the graph (Figure 5(b)) shows increasing tendency as is seen in Figure 4.

 The highest recorded 1-day rainfall values (see Table 1) during the monsoon months of 1961–2005 for the eight stations showed that Valvan, Navaja, and Koynanagar stations have recorded highest 1-day rainfall more than 500 mm which is more than the highest 1-day rainfall at Mahabaleshwar.

## 6. Rain Day and Mean Daily Rainfall Intensity

**Figure 6:**
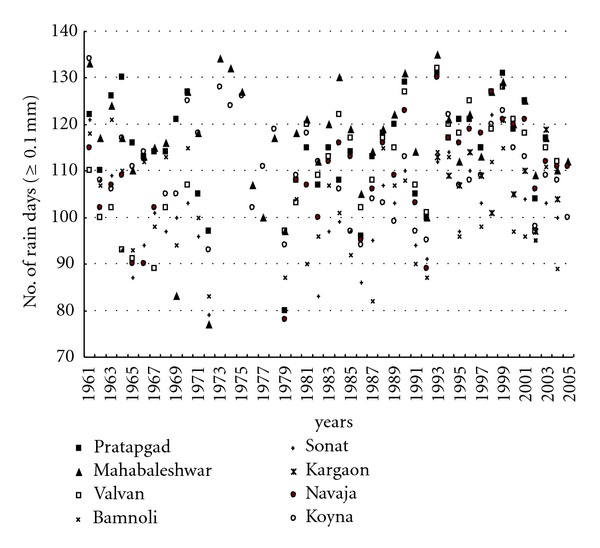
Yearly frequency of number of rain days at eight stations in the Koyna catchment.

**Figure 7:**
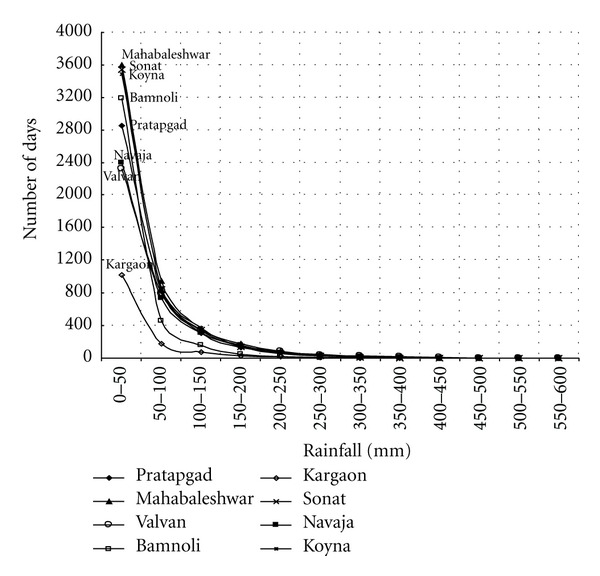
Frequency distribution of daily rainfall over the Koyna catchment.

 The statistical features associated with the daily rainfall distribution, especially during the monsoon season at different stations over a basin, are important aspects of rainfall climatology and interesting for hydropower generation. It is well known that the bulk of the monthly, seasonal, and annual rainfall at a station is contributed by a small percentage of the total number of rain days with large rain amounts. On an average, the number of rain days (i.e., ≥0.1 mm of rainfall) varied from 101 (Sonat) to 116 (Mahabaleshwar) from June to October months of the monsoon season (Table 2).  Figure 6 shows the yearly frequency of rain days for the eight stations in the Koyna catchment, and frequency distribution of daily rainfall is shown in Figure 7.

 A variety of forms of relationship between monthly totals (*T*), rain days (RD), and mean daily rainfall intensity (MDIs) were examined on monthly and seasonal scale. Mean daily rainfall intensities (MDI) were obtained by dividing monthly averages (*T*) by the average number of rain days (RD). It is seen from Table 3 that the lowest intensity (i.e., mean rainfall/day) has been recorded at the Bamnoli station (29 mm/day) situated in the rain-shadow region of the catchment immediately to the east of the Western Ghats, and the highest intensity occurred over the Valvan station (57 mm/day) situated close to the Western Ghats.

 As stated earlier, the CV of the daily rain amounts is a significant parameter of the daily rainfall distribution, which uniquely determines the shape of the NRC. It is found that the CV of the daily rainfall at the 8 stations varies from 110% to 127% and 114% to 129% (see Table 3) during the monsoon months of June to September and June to October, respectively. It is also seen from Table 3 that on an average all the stations recorded more than 100 rain days except the Bamnoli and Sonat stations (95 days) during the monsoon season of June to September.

 Considering the monthly variation, it is seen that CV values range from a minimum of 93.6% for the August rainfall of Navaja to a maximum of 174.1% for October rainfall of Mahabaleshwar. The lowest values of CV are found over the western parts and the southern plain region of the catchment as the daily rain amounts are less variable over these regions. This is mostly due to the combined effect of the monsoon activities in the Arabian Sea and the Bay of Bengal producing heavy to very heavy rainfall over the western region. The largest values of CV are recorded by the stations in the eastern part, falling in the rain shadow region when compared to the western region.

**Figure 8:**
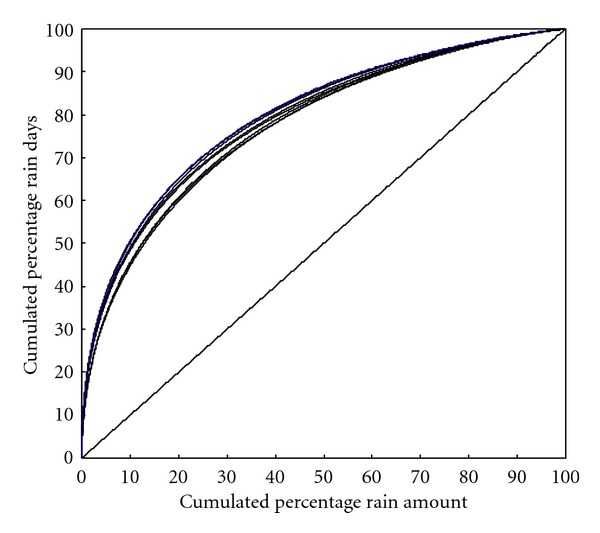
Normalized rainfall curves (NRC) of the stations in the Koyna catchment.

 The analysis also revealed that a large number of rain-days in a season are of low intensity which has contributed only a small fraction to the seasonal total rainfall. On the contrary, a few days of high rain intensity contributed substantial amount of rainfall. This is clear from Figure 8 wherein NRC for all the stations has been shown.

 Since the NRC is directly related to the CV of the rainfall series, the cumulated percentage number of rain days that contribute 50% of the rain amount, calculated from the zero end of the NRC, is directly related to the CV of the rainfall series of the respective stations. It is seen from Figure 8 that high-intensity rain days are clustered around the upper end of the NRC, where the values of cumulative rain amounts and cumulative rain days approach 100%. As the cumulated percentage frequency of rain days (*y*-axis) approaches 100, the slope of the curve tends to zero. All the stations have recorded 50% of the rainfall, calculated from the upper end of the NRC in 13–16% of rain days with heavy falls while the other 50% is contributed by the 84–87% of rain days with falls of low intensity. The 50% of the rainfall contributed by falls of higher intensity in 13–16% of the rain days is more important in recharging the surface water resources, in producing floods, hydropower generation, causing soil erosion, and so forth.

## 7. The Intensities of Rainfall at the Upper End of the NRC

 As mentioned earlier, that high-intensity rain days (see Figure 8) are clustered around the upper end of the NRC, where the values of *x* and *y* approach 100%. The average contribution to the total seasonal rainfall by intensities more than the calculated intensities at *y* = 99% varies from 92.2 to 93.2% for the eight stations whereas in the case of *y* = 99.9% as is expected the values are higher (see Table 4). It is seen from Table 4 that rain intensity is comparatively higher at Valvan and Navaja stations than the Mahabaleshwar station at *y* = 99%. It is also seen that rainfall intensity change on monthly scale.

## 8. Rain Days and Rainy Days

 As stated earlier, the term “rain-day” is used to indicate a day on which a measurable amount of rain, namely, 0.1 mm or more has been recorded at the station. This is different from the India Meteorological Department definition of rainy-days (IMD, 1962). IMD refers to a day with a rain amount of 2.5 mm or more as a “rainy-day.” If we denote these two parameters by *n* and *n*′ for a given station, then it is obvious that *n*′ < *n*. Although, along the Western Ghats, the “rain days” and “rainy-days” are more or less same, the quantity *z* = 100(*n* − *n*′)/*n* for the monsoon season is less than 10% for the stations in the western parts and plain region of the catchment than the stations (especially Bamnoli and Kargaon) in the eastern part of the Koyna catchment.

## 9. Rainy Days and Mean Daily Intensity

In the present study, since the catchment is located in the hilly terrain receiving heavy to very heavy rainfall throughout the monsoon season, average number of rainy days for each of the monsoon month of 1961 to 2005 period for the stations in the Koyna catchment have been calculated using IMD's criteria for rainy day (≥2.5 mm), heavy rainy day (>650 mm) and very heavy rainy day (>1300 mm).  Table 5 gives the average number of rain days for different categories for the Koyna catchment.

**Figure 9:**
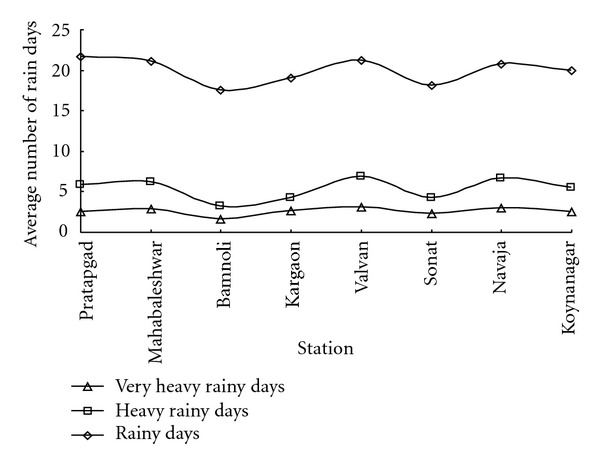
Stationwise distribution of rainy days over the Koyna catchment.

It is seen from this Table 5 that July month has recorded highest number of rainy days in all the three categories followed by August and June months, respectively. This is mostly in relation with the receipt of heavy rainfall during the month of July. The station wise distribution of these rainy days is shown in Figure 9.

The frequencies of heavy and very heavy rainy days recorded by each station in the Koyna catchment are comparatively very low (see Figure 9), varying between the ranges of 2 to 7 whereas the rainy day with 2.5 mm of rain vary from 18 (Bamnoli and Sonat) to 22 (Pratapgad).

 In order to know the rainfall condition over the catchment, the relationship between the seasonal total rainfall, number of rainy days, and mean daily rainfall intensity has been worked out. This may give some indication of frequency of occurrence and broad measure of intensity of rain which are found to be significant from agricultural and hydrological point of view.

The two types of relationship studies have been carried out between

**Figure 10:**
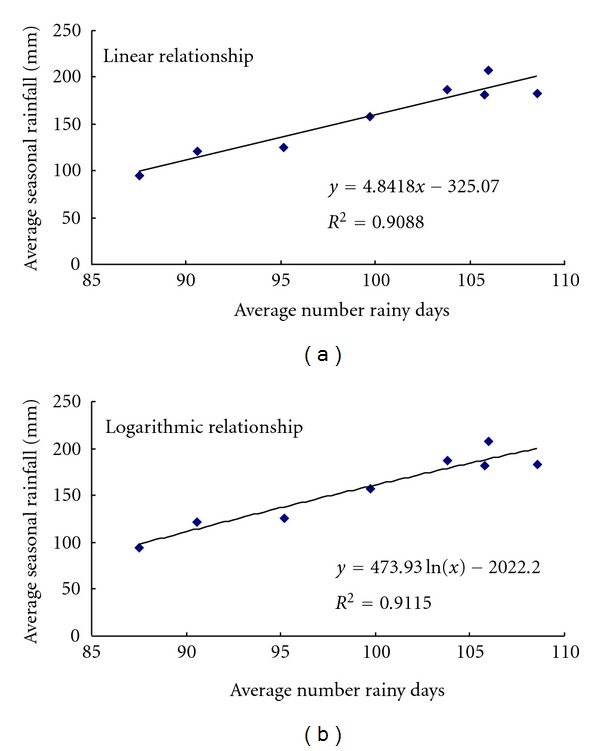
Relationship between Average seasonal rainfall and number of rainy days (a) Linear relationship (b) Logarithmic relationship.

**Figure 11:**
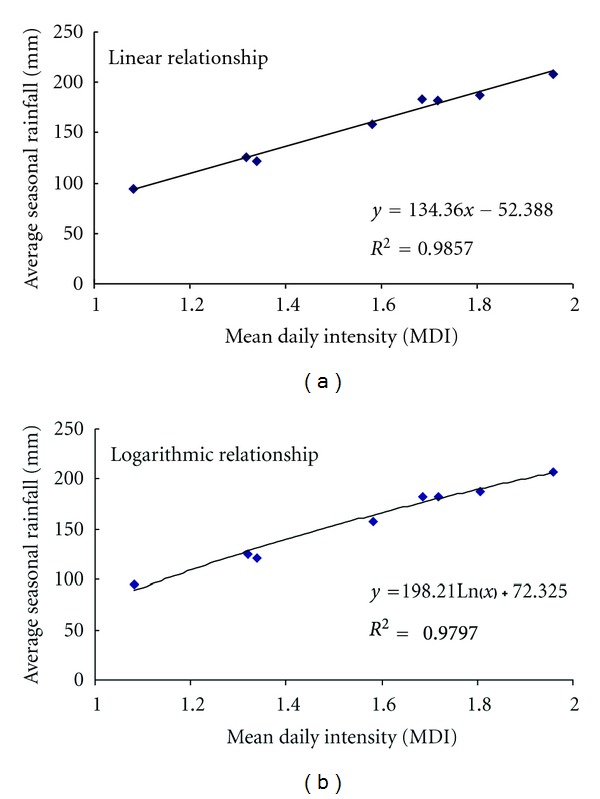
Relationship between Average seasonal rainfall and Mean daily intensity (a) Linear relationship (b) Logarithmic relationship.

- average seasonal rainfall and the number of rainy days (see Table 6 and Figure 10),
- average seasonal rainfall and mean daily intensity (see Table 6 and Figure 11).

Here,

(4) $$\begin{array}{c} \text{Mean}\;\text{daily}\;\text{intensity}=\frac{\text{Average}\;\text{seasonal}\;\text{rainfall}}{\text{Number}\;\text{of}\;\text{rainy}\;\text{days}} \end{array},$$

Table 6 gives the number of rainy days (N.R.D.), mean seasonal rainfall, and mean daily intensity (M.D.I.).

**Figure 12:**
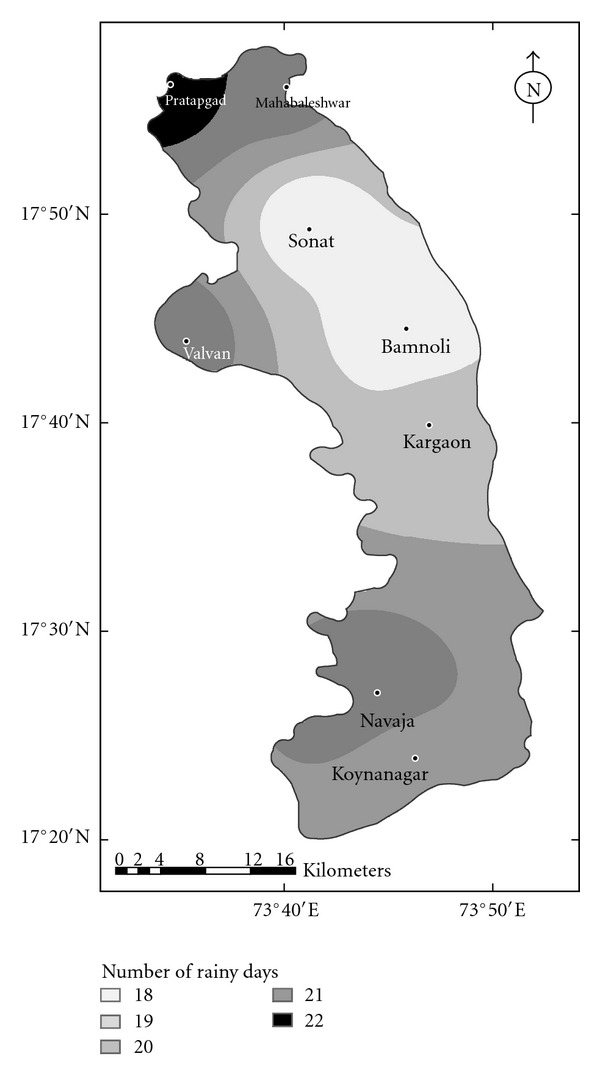
Spatial distribution of rainy days over the Koyna catchment.

It is seen from Figure 10 and mean absolute error analysis (MAE) that, in the case of average seasonal rainfall versus number of rainy days (N.R.D.), although there is a very marginal difference in the *R* ^2^ values, the logarithmic relationship when compared with the linear relationship gave less mean absolute error, and similarly in the case of average seasonal rainfall versus mean daily intensity (M.D.I.) (Figure 11), the logarithmic relationship was found to be better than the linear relationship. *R* ^2^ value was also used to assess the equation (indicated in Figures 10 and 11), and it was seen that differences between the *R* ^2^ values for linear and logarithmic equations were quite small and nonsignificant. Therefore, considering the topography and the heavy rainfall received over the region, logarithmic relationship may be considered as more superior to the linear relationship. The spatial distribution of rainy days over the Koyna catchment is shown in the Figure 12.

## 10. Conclusion

 To summarize it can be said that the Koyna catchment is the major hydroelectric power supplying station in the Maharashtra, India. It is situated in the highly orographic region in the Western Ghats. On the basis of past climatological data, the Koyna Dam was built in 1963, and it always remained in discussion due to seismic activity in nearby vicinity. In view of this, in order to know the rainfall characteristics of the catchment, after the construction of the dam with special reference to climate change has been made in this study. It has been noticed that the stations, Valvan and Navaja, have recorded heavy rainfall compared to Mahabaleshwar rainfall which is considered as the heaviest rainfall receiving station of the catchment. The analysis further shows that mean daily intensity recorded by these two stations is higher than that of the Mahabaleshwar station. The average monthly rainfall for the Mahabaleshwar when compared for the two periods, namely, 1901–1950 and 1951–2005 showed that there is decrease in the average monthly rainfall during 1951–2005.

 The present study deals with the spatial variation of different parameters of the daily rainfall distribution over the Koyna catchment during the southwest monsoon season namely June to October (153 days) and uses daily rainfall data of 8 stations spread more or less uniformly over the catchment for the period of 1961 to 2005. The nature of the normalized rainfall curve (NRC) is uniquely determined by the coefficient of variation (CV) of the rainfall series. The main results and conclusions of the present study are as follows.

1. The mean daily rainfall (*r*) per rain day at the stations during the monsoon season varies from 29 mm/day to 57 mm/day.
2. The CV of the daily rainfall amounts varies from 114% to 129% over the catchment. The maximum values of the CV of the daily rainfall series are found over the eastern part of the catchment.
3. The average number of rain days (*n*) over the catchment varies from 93 to 116 (out of 153 days of June to October); the coefficient of variation of *n* ranges from 6% to 11%.
4. 10–15% of the total number of rain days with heavy falls contributed 50% of the seasonal rainfall at the individual stations.
5. Days of significant rainfall, defined as days in which rain amounts exceed the mean daily rainfall (*r*), constitute about 33% of the total number of rain days and contributed nearly 75% of the seasonal rainfall at almost all the stations.
6. Though the number of rain days exceeds the number of rainy days, there is no large difference between the two as the catchment is situated in the Western Ghat and receives good amount of rainfall during the monsoon season.
7. Between linear relationship and logarithmic relationship number of rainy days versus seasonal rainfall, the logarithmic relationship gave lower mean absolute error (1.83) and hence is superior to linear relationship (1.85).

## Figures and Tables

**Table 1:** Highest 1-day rainfall (mm) for stations in the Koyna catchment.

Station	Highest 1-day rainfall (mm)	Date of occurrence
Pratapgad	482.6	20/07/1982
Mahabaleshwar	490.0	03/08/2004
Bamnoli	310.8	18/08/1991
Kargaon	291.1	12/07/1994
Valvan	509.8	16/07/1965
Sonat	380.0	24/07/1989
Navaja	552.0	25/07/2005
Koynanagar	558.0	26/07/2005

**Table 2:** Average number of rain days ≥ 0.1 mm of rainfall for the stations in the Koyna catchment (1961–2005).

Pratapgad	Mahabaleshwar	Valvan	Bamnoli	Sonat	Kargaon	Navaja	Koyna
114.9	116.5	110.9	102.0	101.2	109.1	109.4	110.6

**Table 3:** Statistical parameters of rainfall series of eight stations in the Koyna catchment.

		Jun	Jul	Aug	Sept	Oct	Jun–Sept	Jun–Oct
Pratapgad	*n*	21.45	29.74	29.58	22.84	8.37	103.61	111.97
	*r*	42.16	71.86	58.5	24.83	18.8	51.53	49.09
	*s*	50.87	70.8	58.89	30.51	26.4	58.95	57.81
	CV	120.65	98.52	100.65	122.84	140.47	114.4	117.76

Mahabaleshwar	*n*	23.47	30.6	29.49	23.04	9.87	106.6	116.47
	*r*	38.74	71.17	58.17	22.98	13.32	50.02	46.91
	*s*	53.29	70.28	57.29	30.56	23.20	59.03	57.78
	CV	137.58	98.76	98.49	132.94	174.10	118.01	123.18

Valvan	*n*	22.26	30.44	30.24	21.00	7.00	103.94	110.94
	*r*	53.49	82.99	66.46	25.43	16.40	60.23	57.46
	*s*	60.33	79.13	66.45	31.95	23.26	67.22	66.19
	CV	112.80	95.35	99.99	125.64	141.81	111.60	115.18

Bamnoli	*n*	18.45	29.16	28.47	18.74	7.16	94.82	101.97
	*r*	28.45	40.91	29.19	13.85	14.04	29.62	28.52
	*s*	36.77	43.92	36.50	17.99	18.17	37.52	36.71
	CV	129.24	107.36	125.04	129.89	129.39	126.68	128.71

Kargaon	*n*	21.00	30.17	28.75	19.83	9.33	99.75	109.08
	*r*	38.20	49.81	36.89	17.67	14.57	37.25	35.31
	*s*	48.19	53.29	42.47	31.29	24.57	46.71	45.68
	CV	126.16	106.97	115.13	177.11	168.67	125.38	129.36

Sonat	*n*	19.51	29.17	28.63	17.6	6.31	94.91	101.23
	*r*	34.06	55.80	38.21	14.57	13.58	38.38	36.83
	*s*	44.20	59.70	44.09	20.19	19.52	48.58	47.67
	CV	129.78	107.00	115.40	138.60	143.80	126.58	129.42

Navaja	*n*	20.94	30.15	29.88	20.91	7.56	101.88	109.44
	*r*	46.71	74.51	62.16	25.93	17.89	55.20	52.62
	*s*	56.23	72.24	58.15	32.87	25.02	60.99	59.96
	CV	120.36	96.96	93.55	126.80	139.91	110.49	113.95

Koyna	*n*	21.96	29.89	29.67	20.78	8.33	102.29	110.62
	*r*	39.93	67.97	48.15	18.91	14.19	46.24	43.82
	*s*	48.01	66.19	48.50	24.55	22.74	53.64	52.64
	CV	120.22	97.39	100.72	129.82	160.19	116.01	120.12

*n*: the mean number of rain days, *r*: the mean rain amount per rain day (mm), *s*: standard deviation (*σ*), and CV: 100 ∗ (s.d./r).

**Table 4:** Estimated daily rain intensities at 99% and 99.9% level for the stations in the Koyna catchment.

Station	Estimated daily rain intensities at	
	99%	99.9%
Pratapgad	93.2	98.8
Mahabaleshwar	92.7	98.8
Bamnoli	92.2	98.7
Kargaon	93.0	99.4
Navaja	93.2	98.7
Koyna	93.1	98.7
Sonat	92.3	98.7
Valvan	93.2	98.9

**Table 5:** Frequency of rainy days from June to October (1961–2005) over the Koyna catchment.

Rain category	June	July	August	September	October
Rainy (≥2.5 mm)	19	29	28	17	7
Heavy (≥65 mm)	4	10	8	2	1
Very heavy (≥130 mm)	2	5	3	2	1

**Table 6:** Number of rainy days (N.R.D.) and mean daily intensity (M.D.I.) and average seasonal rainfall for the stations in the Koyna catchment.

Station name	Rainy days	Ave. seasonal rainfall (mm)	Mean daily intensity (MDI) (mm)
Pratapgad	109	182.90	1.68
Mahabaleshwar	106	181.77	1.72
Bamnoli	88	94.70	1.08
Kargaon	95	125.49	1.32
Valvan	106	207.54	1.96
Sonat	91	121.29	1.34
Navaja	104	187.38	1.81
Koynanagar	100	157.75	1.58
